# A growing degree day model determines the effect of temperature stress on diverse chickpea genotypes

**DOI:** 10.3389/fpls.2024.1496629

**Published:** 2025-02-12

**Authors:** Cara Jeffrey, Laura Ziems, Brent Kaiser, Richard Trethowan

**Affiliations:** ^1^ School of Life and Environmental Sciences, The University of Sydney, Sydney, NSW, Australia; ^2^ The Plant Breeding Institute, The University of Sydney, Sydney, NSW, Australia; ^3^ The Sydney Institute of Agriculture, The University of Sydney, Sydney, NSW, Australia

**Keywords:** chickpeas, abiotic stress, heat, yield, degree days, breeding

## Abstract

Chickpeas are a globally crucial agricultural product, currently at risk due to human-induced climate change. There has been little research into the impact of heat stress on chickpea compared to other crops, but it is known that heat stress can cause up to 100% yield loss. This study measures Growing Degree Days (GDD) in chickpeas, utilizing an existing calculation. This formula has been expanded for heat stress, titled Stress Degree Days (SDD), to examine the effects of high temperature stress on commercially important traits such as yield and seed size. Using a multi-environment trial, traits such as time to flowering, and seed size were observed in 148 chickpea cultivars across two sowing times in two different Australian locations (Narrabri in New South Wales, and Kununurra in Western Australia). It was determined that there is a significant correlation between yield, GDD, and SDD at all locations, sowing times, and life stages of the crop. These metrics allowed greater differentiation between environments when compared to a count of the number of calendar days required for each cultivar to reach a set life stage (flowering and maturation), allowing more accurate investigation the impacts of high temperature stress. It was also determined that loss of yield and a decrease in seed size was significantly correlated with high GDD and SDD, though seed size had less environmental plasticity (variability) compared to yield, and therefore higher stability under stress. GDD and SDD were shown to be useful for predicting genotype adaptation to locations and seasons thus providing a basis for varietal recommendations. This information could also be used to breed environment specific cultivars and to understand trait plasticity.

## 1 Introduction

Chickpea (*Cicer arietinum* L.) is a crucial source of protein and starch in many countries (Abbo et al., 2003), making up roughly 20% of global pulse production (FAO, 2020). Chickpea is adapted to low-nutrient, water-limited conditions, and is able to extract 80% of its nitrogen requirements from soil (Sharma et al., 2013). Nevertheless, abiotic stresses such as drought and heat cause ~70% of global yield losses (Varshney et al., 2019). Human driven climate change remains a critical risk to global agriculture. The global mean departure year (the year when the projected mean climate moves to a state permanently outside historical bounds) is 2069 ± 18, if global emissions are stabilized, and 2047 ± 14 if not (Mora et al., 2013). Previous research has demonstrated that high temperature stress can severely damage chickpea plants, with processes such as photochemical efficiency, pollen viability and germination, and pod set all negatively impacted (Kumar et al., 2013). Improved knowledge of the mechanisms of heat tolerance will assist the genetic improvement of this important crop and its continuation as an important food staple (Blum, 1986).

Growing Degree Days (GDD) is a unit of measurement that relates plant growth and development to air temperature. A degree day is a calculation of the cumulative temperature experienced by a plant at which growth is possible, calculated as GDD = ∑[Tmax + Tmin/2] – Tbase, where (Tmax+Tmin)/2 refers to the average daily temperature, and Tbase is the minimum temperature at which the crop can grow (Mavi and Tupper, 2004).

GDD can be more effective than season length for recognizing physiological responses to temperature (Russelle et al., 1984) and has been used to calculate yield potential in wheat, cowpea, grape, soybean, and tomato (Akyuz et al., 2017; Bondade and Deshpande, 2021; Hall, 2000; Pathak and Stoddard, 2018; Raun et al., 2001). This calculation has also been used to model the growth and development of chickpea, with the earliest study published in 1996 (Singh and Virmani, 1996). Since then, there have been studies examining critical temperatures (Devasirvatham et al., 2012; Sadras and Dreccer, 2015), and phenological variation across varied environments (Dreccer et al., 2018). These studies have been limited in scope due to their use of a small number of genotypes (Dreccer et al., 2018; Sadras and Dreccer, 2015; Verghis et al., 1999), and data collected from simulations (Dreccer et al., 2018; Soltani and Sinclair, 2011). A more recent study has focused on sowing date selection for assurance of healthy growth in chickpea, using thermal sum calculations to optimize sowing time and ensure a healthy growing period devoid of excess heat stress (Richards et al., 2020). In order to strengthen this crop against the impacts of increasing temperatures, it is essential to understand trait plasticity and genetic control among a diverse population under commercial field conditions. This would vastly improve genetic selection and breeding efforts for future cultivars. Similar calculations have been used in chickpea and mung bean to determine methods to breed for cultivars with longer maturation times, to offset maturity acceleration under higher temperatures (Fatima et al., 2021).

GDD is a useful metric with which to evaluate specific crop genotypes for individual farmers, based on their expectation of the season (Hall, 2000). Appropriate application could allow farmers to continuously supply product to market, by choosing genotypes with different GDD requirements and sowing times at different locations (Bondade and Deshpande, 2021). Additionally, the ability to more effectively and accurately align season and harvest expectations could assist in management decisions such as scheduling labor, processing, and long term water management based on season length (Pathak and Stoddard, 2018). In this study, the calculation was modified to include Stress Degree Days (SDD), which refers to the cumulative temperature experienced by plants exposed to temperature stress. This stress is recorded here as a decrease in yield or seed size, as per existing literature (Davies et al., 1999; Kumar et al., 2013; Zinn et al., 2010). In this paper, stressful temperatures are considered to be above 30°C as per existing literature (Sleimi et al., 2013).

This study uses GDD to evaluate 148 genotypes in two contrasting environments to select genotypes based on their performance and phenotypic plasticity. These were; a tropical environment that experiences consistent high temperatures but is otherwise “ideal” due non-limited water availability and light conditions, and a more variable, temperate environment considered more typical to the “Mediterranean” type for which Chickpea was domesticated (Abbo et al., 2003). SDDs were calculated to understand the impact of cumulative heat-related stress on the crop to determine both genetic and environmental effects on the traits assessed. Better understanding the response of genotypes to varying climates provides a critical tool that farmers can use to improve and secure harvest outcomes.

## 2 Materials and methods

### 2.1 Data collection – field trials

Two Australian locations with varying climates were chosen to collect physiological data. The first was in Kununurra, Western Australia (15.7783°S, 128.7439°E), the second in Narrabri, New South Wales (30.2737°S, 149.7350°E). The recorded minima and maxima temperature for these locations are shown in Appendix 1, and these data were collected from stations managed by ozforcast.com.au and weather.agric.wa.gov.au, respectively. In summary, for the purpose of this trial, Kununurra is considered an environment in which the trials experienced only high temperature stress, as the trials were flood irrigated and regularly treated with nitrogen. Due to this, the Kununurra site could be considered an “ideal” environment when studying heat stress, as other confounding stressors are absent. Narrabri can be considered a more typical environment for chickpea production, experiencing the milder mid-season temperatures, with the crop receiving rain and supplementary irrigation as required.

Field experiments were conducted at these locations in 2019, at two sowing times. Experiment refers to the year and environment in which the trial was executed. The first time of sowing (TOS) was based on typical commercial growing times within each region, and the second was delayed so that flowering would occur when average temperatures were higher, thus generating heat stress from reproductive development onwards. In Kununurra, this delay was 1 month, as the higher temperatures caused a shorter season length. In Narrabri it was 2 months, due to the milder conditions. This resulted in a total of 4 experiments across 2 environments and 2 TOS.No soil analyses were conducted as the focus was on the temperature stress (manipulated through location and time of sowing). Experimental plots were located in the same field with the same cropping history/agronomic practices minimizing soil variability.

The 148 elite chickpea genotypes evaluated included 10 Desis and 138 Kabulis, originating from Syria (129), India (10), and Australia (9) (Appendix 2). Those genotypes not from Australia were provided by ICARDA and ICRISAT and were considered high yielding in hot environments. Australian genotypes were chosen as comparison, being already used in Australian environments. Genotypes were sown according to commercial standards (~169 seeds and ~381 seeds per plot in Narrabri and Kununurra respectively) in randomized complete block designs of 2 replicates for each TOS in plots of 8 m^2^. Randomized complete block designs were used to control for variability across the field. Differences between replicates were considered through randomized complete block design and treating replicates as random effects. Main effects of genotype and environment (location and time of sowing) were significant, while differences between replicates were inconsequential (**Tables 1**–**3**). Each trial was prepared and treated using standard agronomic practice including fertilizer, herbicide and pesticide recommendations at each location (Appendix 3). Plots were inspected daily to identify date of flowering and maturity. This was recorded as the number of days from sowing to each stage of development. According to standard commercial practices, the growth season for Chickpea in Kununurra is typically shorter than in Narrabri (GRDC, 2016; Regan, 2015). All plots were harvested when the entire experiment attained harvest ripeness. Gross yield and 100 seed weight (HSW) was assessed in grams post-harvest. Weather data was collected using an on-site weather station which sampled every 15 minutes. This was collated into daily averages across the season. Across all trials, data can be categorized into plant physiological and climate data. Plant physiological data comprised number of days to flowering, and maturity, the weight of 100 seeds in grams, and gross plot yield in grams. Climate data refers to the temperature regimes at each location over the duration of the trial.

**Table 1:** Superiority and Static stability coefficients for 100 seed weight (HSW) and yield depicting the 10 highest ranked genotypes for each trait and metric.

		Superiority		Static Stability	
	Rank	Narrabri	Kununurra	Narrabri	Kununurra
HSW	1	* ^A^ 139	* ^A^ 139	38	89
	2	*1	*138	109	11
	3	*88	*88	34	**68
	4	60	22	10	71
	5	*75	*21	12	58
	6	*138	*75	122	^D^ 141
	7	61	*53	**39	22
	8	127	137	82	*100
	9	*53	98	70	5
	10	*21	*1	*100	^D^ 133
Yield	1	* ^D^ 142	^A^ 146	24	97
	2	*70	* ^A^ 145	30	127
	3	^A^ 131	*142	69	77
	4	* ^A^ 145	40	**39	92
	5	36	114	78	54
	6	47	*70	120	33
	7	^A^ 143	115	46	75
	8	76	128	88	67
	9	62	37	3	**68
	10	^D,A^ 140	116	116	^D,A^ 140

Genotypes denoted with * have high ranking for the trait in both environments, and those denoted with ** have high rankings for both traits in that environment. Superscript D refers to genotypes that are Desi type, and superscript A refers to Australian genotypes.

**Table 2:** Summary of average number of days to reach each life stage (Age), average number of days with temperature suitable for growth (# Days), average growing degree days (GDD), average stress degree days (SDD), average yield, and average 100 seed weight (HSW) for each time of sowing (TOS) and location.

Flowering				Maturity				Yield (T/ha)	HSW (g)	TOS	Trial
Age	# Days	GDD	SDD	Age	# Days	GDD	SDD				
85 ± 5	62	182 ± 20	0 ± 1	134 ± 2	111	511 ± 18	13 ± 2	2.23 ± 0.39	35.16 ± 5.29	1	Narrabri
65 ± 3	58	281 ± 34	5 ± 3	102 ± 2	95	673 ± 23	25 ± 2	1.15 ± 0.30	31.57 ± 5.15	2	
57 ± 9	54	783 ± 105	118 ± 11	128 ± 3	125	1561 ± 114	216 ± 13	3.51 ± 0.69	36.95 ± 6.34	1	Kununurra
55 ± 7	55	641 ± 71	76 ± 7	131 ± 6	131	1591 ± 109	246 ± 24	3.3 ± 0.58	39.04 ± 6.68	2	

Flowering is determined as the date at which ≥ 50% of plants have ≥ 1 flower, maturity is determined as the date at which ≥ 50% of plants are ≥ 50% desiccated.

**Table 3:** Spearman’s rank correlation between Growing Degree Days (GDD) and Stress Degree Days (SDD) of two growth periods with grain yield and 100 seed weight (HSW) at each location.

Location	Growth Period	Variate 1	Variate 2	Adjusted R^2^
Kununurra	Vegetative	GDD	HSW	0.041
			Yield	*-0.18
		SDD	HSW	-0.082
			Yield	**-0.198
	Reproductive	GDD	HSW	0.015
			Yield	*0.183
		SDD	HSW	*0.164
			Yield	**0.187
Narrabri	Vegetative	GDD	HSW	**-0.29
			Yield	**-0.807
		SDD	HSW	**-0.333
			Yield	**-0.489
	Reproductive	GDD	HSW	*-0.182
			Yield	**-0.615
		SDD	HSW	**-0.306
			Yield	**-0.802

Vegetative is defined as the period between sowing and flowering, and Reproductive as the period between flowering and physiological maturity. * and ** denote significant (p <0.05) and highly significant (p < 0.001) correlations respectively.

### 2.2 Data analysis

#### 2.2.1 Physiology and environment

Preparation of the data, and other relevant statistical tests were completed using Genstat (VSN-International, 2022). Genotypes and times of sowing were considered fixed effects at each location in each year, and rows and ranges within replications and times of sowing as random effects. Predicted means were calculated for further analysis. Correlations between the generated GDD and SDD data and the crop physiological traits, and their significance, were assessed using the Spearman’s rank correlation coefficient. Predicted means were used for both yield and related physiological traits.

GDD was calculated from sowing to each key life stage using the R package “cropgrowdays” (https://cran.r-project.org/package=cropgrowdays). The base temperature was set at 10°C (Tmin), and a chosen thermal maximum at 30°C (Tmax), in accordance with existing literature (Sleimi et al., 2013), to ensure the calculation only accounted for temperatures at which growth was optimal. SDD was calculated by repeating this script, with the thermal maximum set to 50°C, then subtracting the original GDD value(Formula 1). Subscripts refer to the selected thermal maximum).


(1) $$\text{SDD}={\text{GDD}}_{50}-{\text{GDD}}_{30}$$

(2) GDD=∑[Tmax+Tmin/2]–Tbase

50°C was chosen as it was deemed higher than the recorded maximum temperature experienced in all experiments, thus isolating all days above 30°C for the calculation. Genotypes are labelled numerically for simplicity (Appendix 2), and a summary of the temperature regimes recorded in each experiment is given in Appendix 1.

Superiority refers to the performance of each genotype for the given trait, compared to the others in the population. The stability coefficient refers to variance among means in various environments (Lin and Binns, 1988; Nassar and Huehn, 1987). Superiority coefficients are a calculation of the cultivar superiority measure described in Lin and Binns (1988). Static stability coefficients refer to the variance between means in various environments, which provides a measure of genotypic consistency (VSN-International, 2022). Superiority and static stability coefficients were also calculated using the Meta-Analysis suite found in Genstat by grouping data by genotype and TOS. One calculation was completed for each location, with Y-variate and Environment represented by the assessed trait and TOS respectively, and data was then sorted according to the ranks of each trait’s superiority score (**Table 1**), and then according to the ranks of the Static Stability (**Table 1**). Those that were ranked 10^th^ or lower were selected to highlight in **Table 1**.

#### 2.2.2 Genotypes

GGE biplots were constructed using the Genstat Meta-Analysis suite, with data grouped by both year and location alongside genotype. GGE biplots are multivariate PCA biplots that represent genetic versus phenotypic plasticity of a particular trait among a range of genotypes and environments. Plasticity refers to the ability of the plant to change in response to certain stimuli, such as heat (Scheiner, 1993). PC refers to the principal component, with PC1 representing the maximum variance direction in the data. The origin of each individual graph represents a calculated “virtual” genotype with average performance or broad adaptation across environments, and genotypes that fall closer to a specific environmental vector perform better for that trait in that environment. The angle between environmental vectors indicates the degree of correlation between environments, with a smaller angle denoting greater similarity (Simon Harding, 2010). The choice to use GGE biplots was based on their creation of a number of “virtual” genotypes in order to observe their performance across the various environments, thus examining their plasticity when exposed to different stimuli.

Broad sense heritability was calculated using the equation *H* ^2^ = (*s_g_* ^2^)/[*s_g_* ^2^ + (*s_e_* ^2^/*r*)], in which *s_g_* ^2^ represents the genetic variance, *s_e_* ^2^ represents residual variance, and r represents the number of genotype replicates (Gitonga et al., 2014). Heritability was calculated using predicted means within sites, creating a within-environment model. This means there was one model created per environment. The genetic and residual variance were calculated using a Linear Mixed Model in Genstat, in which the fixed and random models were experiment and genotype respectively, and the Y-variate was the assessed trait.

## 3 Results

### 3.1 Environment and physiology

There was greater range and variability in the SDD and GDD data when compared to Grow Days (**Table 2**). This was particularly notable when considering the difference in grow days between locations for maturity, compared to their respective GDD and SDD values. The GDDs for Kununurra at maturity were triple those of Narrabri, and the SDDs tenfold, whereas the grow days (# Days) were 30% higher at most. Additionally, there was a greater proportional difference between flowering GDD at Narrabri (TOS2 was 35.3% larger than TOS1), than between TOS in Kununurra (TOS2 was 18.1% larger than TOS1). There was greater variation for age at flowering between TOS in Narrabri (23.5%) compared to Kununurra (3.5%), and both TOS in Kununurra reached flowering in a much shorter time compared to Narrabri (32.9% and 15.4% faster, respectively). However, the time between flowering and maturity was shorter for both TOS in Narrabri (49 and 37 days, respectively) compared to Kununurra (71 and 76 days, respectively).

A large difference in yield was observed between locations, with mean yield at Kununurra (x = 3.4 T/ha) 50% higher and HSW (x = 38.0 g) 12.1% higher than Narrabri. However, there was less variation between TOS in Kununurra for both yield (6%) and HSW (5.4%), than Narrabri (48.5% and 10.2% respectively). There was also less variation for HSW than Yield, (19.2% and 67.3% respectively) across all experiments.

GDD and SDD are predominately significantly correlated with HSW throughout the lifespan of the crop (**Table 3**). This correlation was mostly negative, particularly at Narrabri (R^2^ = -0.333, -0.29, -0.182, P < 0.001, P < 0.001, P = 0.002), though the SDD value in Narrabri was consistently much lower than Kununurra (**Table 2**). However, there was a significant positive correlation between HSW and SDD observed during the reproductive stage in Kununurra (R^2^ = 0.164, P = 0.005). There was no significant correlation between GDD and SDD for HSW in the vegetative period at Kununurra (P = 0.479, P = 0.158), nor was there for GDD during the reproductive period (P = 0.802).

From **Table 3**, it can be noted that both GDD and SDD are highly significantly correlated with Yield (P < 0.05): particularly in Narrabri (P < 0.001). This correlation is exclusively negative in the vegetative growth period (R^2^ from -0.807 to -0.180) but positive in Kununurra during the reproductive period, with a stronger correlation between yield and SDD (R^2^ = 0.187, P < 0.001) than GDD (R^2^ = 0.183, P = 0.002).

**Figure 1:**
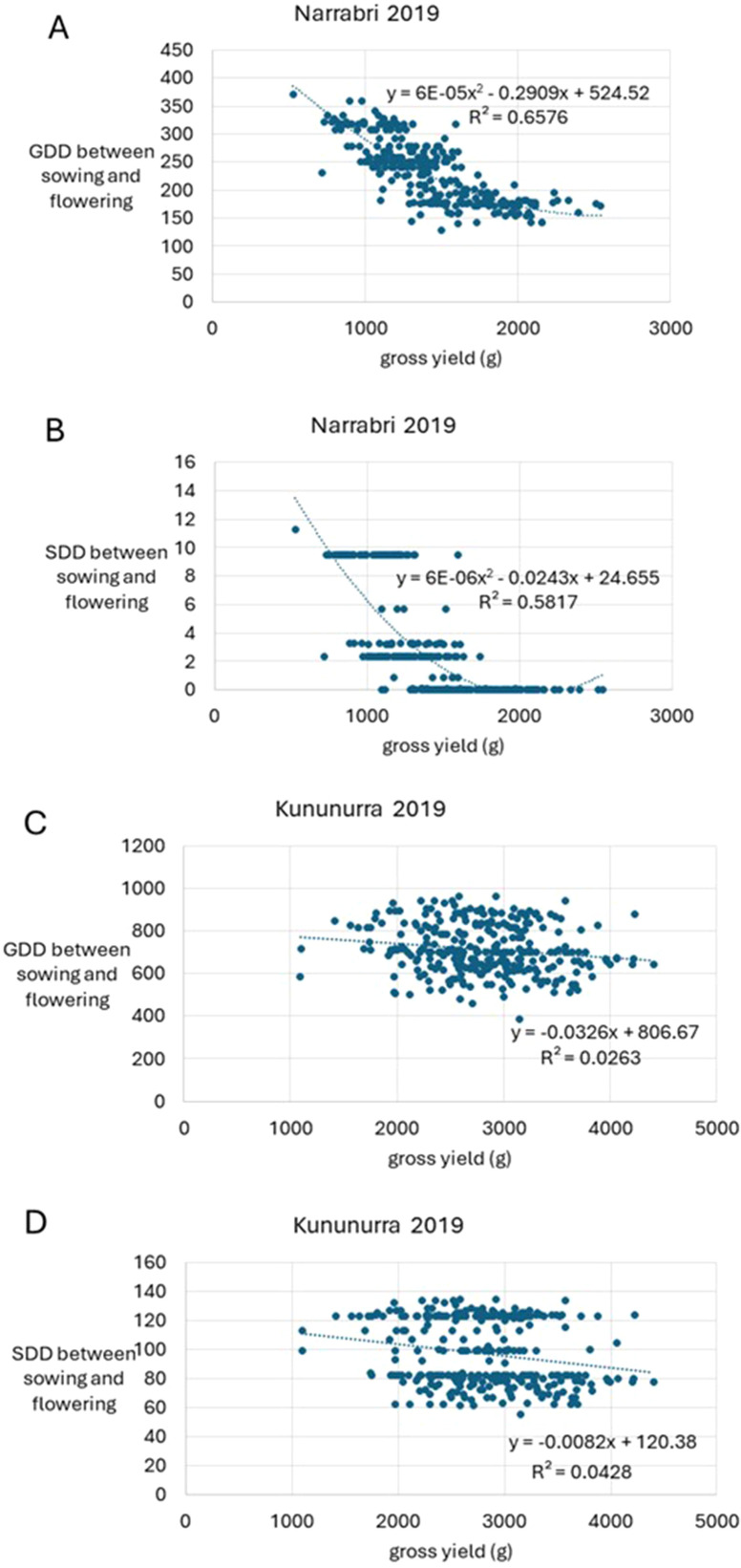
Graphs depicting trends between growing degree days (GDD), stress degree days (SDD), and gross yield (grams) within two separate environments (Narrabri NSW and Kununurra WA) in 2019. Flowering refers to the date at which 50% of the plants in the plot have at least one flower. Graphs have been labelled **(A-D)**, according to their location and whether they depict GDD or SDD data.

**Figure 1** continues to show the negative correlation between GDD, SDD and yield between sowing and flowering. This trend is strongest in Narrabri for both SDD and GDD compared to Kununurra, with negative exponential trends and higher R^2^ values. For Narrabri, the trend between GDD and yield is stronger than SDD and yield (R^2^ = 0.66 and 0.58 respectively), whereas for Kununurra, the reverse is true, with SDD having a stronger negative correlation compared to GDD (R^2^ = 0.04 and 0.03 respectively) (**Figure 1**).

### 3.2 Trait plasticity

**Figure 2:**
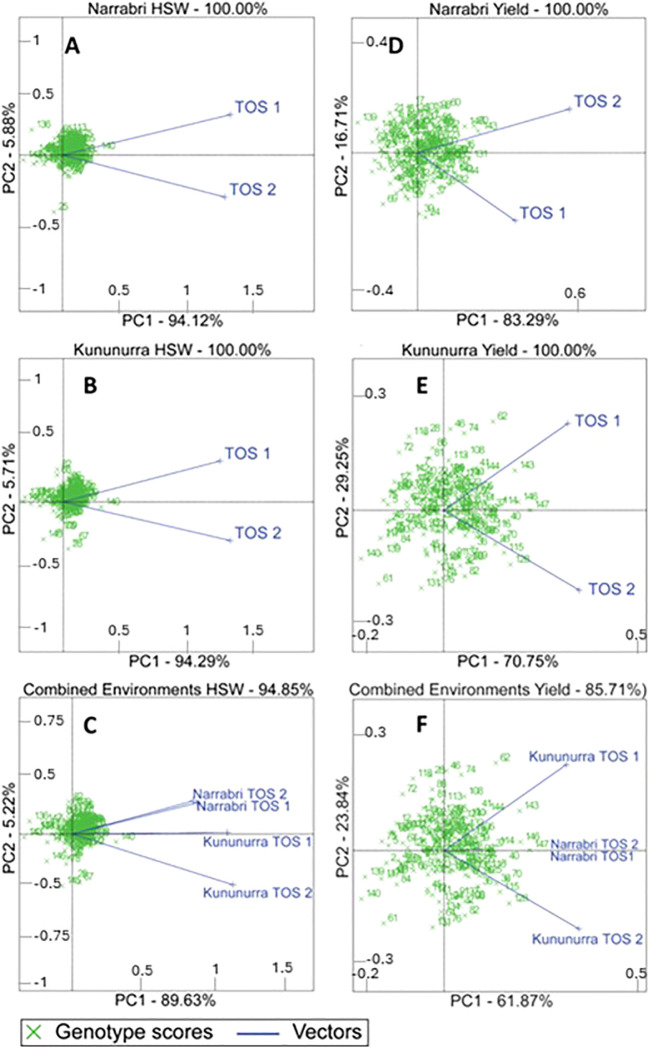
Biplots comparing the Genotype x (Genotype x Environment) (known as GGE) effects across all genotypes, in each environment and combined across environments for grain yield and HSW (100 seed weight). PC1 and PC2 refer to the percentage of variance explained by the genotype and environment respectively. Numbers refer to individual genotypes, whose official titles and countries of origin can be found in **Appendix 1**. **(A)** refers to Narrabri HSW, **(B)** refers to Kununurra HSW, **(C)** refers to the combined environments HSW. **(D)** refers to Narrabri total yield, **(E)** refers to Kununurra total yield, and **(F)** refers to the combined environments' total yield.

The GGE effects of each environment on yield and HSW are given in **Figures 2A–F**. HSW was more influenced by genotype than environment, evidenced by high PC1 scores (89.63 – 94.29%) and acute angles between each environmental vector. Genotypes that clustered towards the origin demonstrated a similar performance to the virtual genotype.

Yield was impacted more significantly by the environment in comparison to HSW. There are wider angles between environmental vectors, and lower PC1 scores (61.87 – 83.29%). Genotypes are spread further from each other, with some genotypes associated with specific environmental vectors.

Variance in yield at Narrabri was more strongly related to genotype (83.29%) than Kununurra (73.25%). In Kununurra, a larger number of genotypes aligned more closely with specific environmental vectors than Narrabri, demonstrating superior performance in this environment.

For HSW, 60% of top performers were common to both environments, and 10% were top performers or highly stable across traits in each environment (**Table 1**). For yield, 30% of top performers were common to both environments and 10% were highly stable (**Table 1**). In terms of consistent stability across traits, genotypes 39 and 68 had high stability for both HSW and yield in Narrabri and Kununurra (**Table 1**).

No genotypes were both high performers and highly stable in each environment.

Desi genotypes represent 6.76% of the genotypes assessed in these experiments but represented 30% of the genotypes ranked in the top 10 for yield (**Table 1**). Desi genotypes represented 20% of the genotypes ranked in the top 10 for HSW stability, but these genotypes were only considered highly ranked based on performance in Kununurra (**Table 1**).

### 3.3 Heritability

HSW had a higher heritability than yield overall (H^2^ = 0.916 – 0.970 and 0.378 – 0.956, respectively). HSW had a more consistent heritability estimated across environments when compared to yield (3.6% and 60.4% variation, respectively). HSW had a more consistent heritability estimate in Narrabri compared to Kununurra (0.2% and 4.6% variation respectively), whereas yield showed consistently lower heritability in Narrabri compared to Kununurra (17.9% and 4.3% variation respectively). The experiment with the highest heritability across both traits was Narrabri TOS 2, and the lowest was Kununurra TOS 2.

## 4 Discussion

### 4.1 Climate and physiology

This study showed that GDD and SDD provide greater variation in field data compared to a simple count of the number of days that were at suitable temperature for growth. Data for both geographical locations were vastly different for each calculation, however this was to be expected, considering the vast differences overall climate (Appendix 2), and the differing agronomy practices at each location, such as more extensive watering at Kununurra. However, this variation allowed detailed investigation of genotypic and environmental influences on key traits (**Figure 2**, **Table 2**), due to the bespoke environment of each trial. This diversity of conditions highlights the need to develop location-specific cropping strategies and genotypes for farmers. This study helps provide the ability to develop these strategies with the capacity to predict the impact of climate on yield potential, given a single year of data (**Figure 1**; **Table 3**).

Both TOS in Kununurra reached flowering sooner than Narrabri, likely due to a larger accumulation of GDD in Kununurra over both TOS (**Table 2**). Additionally, yield in Kununurra was 50% higher on average than Narrabri (**Table 2**). This high GDD indicates a consistently warm environment, with reduced vegetative development and earlier flowering. Furthermore, several studies have shown a correlation between early flowering and high yield (Kumar et al., 2013; Rubio et al., 2004; Siddique et al., 2003). Apart from higher GDD, Kununurra also had higher SDD across the season compared to Narrabri. As the SDD calculation refers to the stress experienced at this location, one may expect a decrease in yield in Kununurra compared to Narrabri. However, commercial irrigation standards in Kununurra are considerably more generous, thus plants had greater capacity for stress-management via transpiration cooling, compared to Narrabri, where trials were irrigated according to typical rainfed conditions for the region. Previous research on the impacts of temperature and water stress on chickpea, found that sites with high rainfall typically demonstrated high yield performance (Dreccer et al., 2018), and this was likely due to the plant’s ability to reduce canopy temperatures by 6-10°C (Mathur et al., 2014). Interestingly, HSW in TOS2 was also 5.4% larger than TOS1 in Kununurra (**Table 2**), likely due to a longer period between flowering and maturity. This longer grain filling period results in larger seeds (Davies et al., 1999; Zaiter and Barakat, 1995), as well as the consistent development of new seeds/pods due to the indeterminate nature of the species (Singh, 1997). There is a delicate balance here, as it has been shown that in Australian conditions, early maturing chickpeas with limited water have higher yields only when temperatures are optimal (Sabaghpour et al., 2003). In this study, SDD and GDD were both negatively correlated with yield throughout the season in Narrabri, particularly in TOS2 (**Figure 1**; **Tables 2**, **3**). Thus, it can be seen that even though SDD was lower than Kununurra, stress management is often reliant on resource availability (Mathur et al., 2014).

### 4.2 Trait plasticity

#### 4.2.1 Seed size stability

HSW showed less variation than yield across environments across TOS (**Table 2**). Multivariate analysis showed that HSW was more strongly influenced by genotype than the environment, evidenced by high PC1 scores (89.63 – 94.29%) and acute angles between each environmental vector (**Figures 2A–C**; **Table 2**). Stability coefficients for HSW showed that 60% of the top-ranked genotypes occurred in both locations, compared to 30% for yield, and correlations between GDD and SDD for HSW were lower than observed for yield (**Table 3**). Seed size was also found to be 10.15% and 58.57% more heritable than yield in Narrabri and Kununurra respectively (**Table 4**). These data confirm that yield is an environmentally plastic trait, whereas HSW is more genotypically fixed (**Figure 2**; **Table 2**) and therefore stable. Others reported that seed size in chickpea is controlled by two genes with dominance epistasis (Upadhyaya et al., 2006) and by additive effects (Upadhyaya et al., 2011) and molecular markers directly related to seed size have also been identified (Cho et al., 2002; Cobos et al., 2007; Jamalabadi et al., 2013). These key genetic loci are likely to be present among the genotypes in this study, given that seed size was clearly under relatively strong genetic control. Whereas yield is known to be significantly influenced by environment and therefore less stable (Millan et al., 2006). Several other relevant genetic loci have recently been found related to seed size and yield, which could be used alongside these calculations for breeding improved cultivars (Jeffrey et al., 2024).

**Table 4:** broad sense heritability estimates (H^2^) across location and time of sow (TOS) for 100-seed weight (HSW) and gross yield.

Location	TOS	HSW H^2^	Yield H^2^
Narrabri	1	0.970	0.785
	2	0.968	0.956
Kununurra	1	0.950	0.395
	2	0.916	0.378

#### 4.2.2 Room for enhancement with yield plasticity

One of the benefits of environmental impacts on yield is the option to breed specifically adapted cultivars once the degree of yield plasticity is known. **Figure 2** demonstrates the possibility of choosing genotypes specifically adapted to each environment type. **Figures 2D–F** show a greater spread of data points across the biplot, along with lower PC1 scores compared to those of A, B, and C. It is therefore possible to identify genotypes that are better adapted to TOS2, where temperature stress is likely to be higher. This is particularly evident in Kununurra (**Figure 2E**; **Table 2**), which could be a consequence of the transpiration cooling mentioned previously (Mathur et al., 2014).

In Narrabri, variance in yield was more strongly related to genotype (83.29%) than Kununurra (73.25%) (**Figures 2D, E**; **Table 2**), which is also supported by the higher heritability of yield at Narrabri compared to Kununurra (x = 0.871 and 0.387 respectively) (**Table 4**). Several genotypes in plot E align more closely with one specific vector, demonstrating more variation in genotype performance at each TOS in this location (**Figure 2**; **Table 2**). Clearly, some genotypes are better adapted to TOS2 than TOS1 for yield (and vice-versa). For example, in Narrabri, genotypes 38, 144, and 142 appear to have superior yield in TOS2, whereas genotypes 70, 146, and 143 have higher yield in TOS1 (**Figure 2D**; **Table 2**). Similarly, in Kununurra, genotypes 38, 115, and 128 appear to have superior yield in TOS2, whereas genotypes 105, 143, and 144 were higher yielding in TOS1 (**Figure 2E**; **Table 2**). Understanding these relationships provides a basis for future chickpea improvement, as parents can be selected to either breed for broad adaption or specific adaptation to particular environment types.

### 4.3 Future directions and breeding

Australian genotypes represented 5 of the 10 highest yielding genotypes with highest HSW across environments HSW (**Table 1**). However, in terms of static stability, only one genotype was represented, ranking 10^th^ in yield, and this was predominately observed only at Narrabri (**Table 1**). This suggests that the Australian genotypes are well adapted to this environment, although the average yield loss between Narrabri times of sowing was 32.34% (**Table 2**). It can therefore be concluded that the heat tolerance of Australian cultivars could be improved by utilizing some of the international genotypes that ranked highly in static stability as parents (**Table 1**; **Appendix 1**).

There is clearly a trade-off between seed size and seed number with consequences for yield. However, this allows for tailoring of the product to several markets. Farmers will be driven by price. If gross margins are greater for high yield, then smaller seeded types will be produced. However, a premium price for large seed may change this equation. Unfortunately, no genotypes that ranked highly for both stability and yield. However, some genotypes had both HSW and yield in specific environments, suggesting these may be good choices as breeding stock. Due to the vast differences in environments both between and within locations, it would be unlikely that high performers for either trait would also be stable. However, stable genotypes (**Table 1**) may be a good choice for farmers in highly variable environments with unpredictable seasons.

## 5 Conclusion

The GDD metric calculation is a viable and highly effective tool for assessing the impact of temperature regimes on commercially cultivated chickpeas. With the addition of SDDs, it is possible to further examine the effects of temperature stress on commercially important traits such as yield and seed size and potentially predict the impacts that predicted climate will have on these traits. These models could be further improved with use on literature data for granularity. As risks related to temperature stress increase globally, it is crucial to rapidly find new ways to model and improve genotypes to safeguard food production. This study demonstrated there is a significant correlation between heat stress and yield loss, as well as heat stress and a decrease in seed size. However, seed size was found to be impacted significantly less by environmental factors than yield and largely related to genotype. With these findings, these calculations could be used by breeders to further inform efforts to improve heat tolerance in legume crops such as chickpea, or to prepare for high temperature seasons by choosing specific genotypes. This research provides a basis for selecting chickpea genotypes adapted across environments and/or adapted to specific environment types including high temperatures.

## Data Availability

The raw data supporting the conclusions of this article will be made available by the authors, without undue reservation.
