# Supplementary material for: A growing degree day model determines the effect of temperature stress on diverse chickpea genotypes
Source: Front Plant Sci. 2025 Feb 12;15:1496629. doi: 10.3389/fpls.2024.1496629 (PMC11861096; doi:10.3389/fpls.2024.1496629)
Supplement: Supplementary file 1 [file Table1.docx]

**Appendix 1**. Recorded season temperature maxima and minima for the trial in both Narrabri and Kununurra, collected from stations managed by ozforcast.com.au and weather.agric.wa.gov.au respectively

**Appendix 2**. Genotypes used in this study, organised alphabetically and numerically, with their type and country of origin labelled.

| # | Genotype | Type | Origin |
| --- | --- | --- | --- |
| 1 | Almaz | Kabuli | Syria |
| 2 | FLIP 07-291C | Kabuli | Syria |
| 3 | FLIP 07-295C | Kabuli | Syria |
| 4 | FLIP 07-306C | Kabuli | Syria |
| 5 | FLIP 07-312C | Kabuli | Syria |
| 6 | FLIP 07-328C | Kabuli | Syria |
| 7 | FLIP 07-329C | Kabuli | Syria |
| 8 | FLIP 07-339C | Kabuli | Syria |
| 9 | FLIP 07-340C | Kabuli | Syria |
| 10 | FLIP 09- 84C | Kabuli | Syria |
| 11 | FLIP 09- 90C | Kabuli | Syria |
| 12 | FLIP 09-125C | Kabuli | Syria |
| 13 | FLIP 09-126C | Kabuli | Syria |
| 14 | FLIP 09-129C | Kabuli | Syria |
| 15 | FLIP 09-133C | Kabuli | Syria |
| 16 | FLIP 09-136C | Kabuli | Syria |
| 17 | FLIP 09-140C | Kabuli | Syria |
| 18 | FLIP 09-141C | Kabuli | Syria |
| 19 | FLIP 09-146C | Kabuli | Syria |
| 20 | FLIP 09-148C | Kabuli | Syria |
| 21 | FLIP 09-150C | Kabuli | Syria |
| 22 | FLIP 09-154C | Kabuli | Syria |
| 23 | FLIP 09-155C | Kabuli | Syria |
| 24 | FLIP 09-156C | Kabuli | Syria |
| 25 | FLIP 09-157C | Kabuli | Syria |
| 26 | FLIP 09-158C | Kabuli | Syria |
| 27 | FLIP 09-159C | Kabuli | Syria |
| 28 | FLIP 09-160C | Kabuli | Syria |
| 29 | FLIP 09-164C | Kabuli | Syria |
| 30 | FLIP 09-165C | Kabuli | Syria |
| 31 | FLIP 09-168C | Kabuli | Syria |
| 32 | FLIP 09-169C | Kabuli | Syria |
| 33 | FLIP 09-170C | Kabuli | Syria |
| 34 | FLIP 09-173C | Kabuli | Syria |
| 35 | FLIP 09-179C | Kabuli | Syria |
| 36 | FLIP 09-180C | Kabuli | Syria |
| 37 | FLIP 09-183C | Kabuli | Syria |
| 38 | FLIP 09-184C | Kabuli | Syria |
| 39 | FLIP 09-185C | Kabuli | Syria |
| 40 | FLIP 09-186C | Kabuli | Syria |
| 41 | FLIP 09-187C | Kabuli | Syria |
| 42 | FLIP 09-193C | Kabuli | Syria |
| 43 | FLIP 09-194C | Kabuli | Syria |
| 44 | FLIP 09-201C | Kabuli | Syria |
| 45 | FLIP 09-202C | Kabuli | Syria |
| 46 | FLIP 09-203C | Kabuli | Syria |
| 47 | FLIP 09-207C | Kabuli | Syria |
| 48 | FLIP 09-208C | Kabuli | Syria |
| 49 | FLIP 09-209C | Kabuli | Syria |
| 50 | FLIP 09-213C | Kabuli | Syria |
| 51 | FLIP 09-214C | Kabuli | Syria |
| 52 | FLIP 09-216C | Kabuli | Syria |
| 53 | FLIP 09-218C | Kabuli | Syria |
| 54 | FLIP 09-219C | Kabuli | Syria |
| 55 | FLIP 09-234C | Kabuli | Syria |
| 56 | FLIP 09-263C | Kabuli | Syria |
| 57 | FLIP 09-269C | Kabuli | Syria |
| 58 | FLIP 09-277C | Kabuli | Syria |
| 59 | FLIP 09-280C | Kabuli | Syria |
| 60 | FLIP 09-284C | Kabuli | Syria |
| 61 | FLIP 09-300C | Kabuli | Syria |
| 62 | FLIP 09-308C | Kabuli | Syria |
| 63 | FLIP 09-320C | Kabuli | Syria |
| 64 | FLIP 09-321C | Kabuli | Syria |
| 65 | FLIP 09-322C | Kabuli | Syria |
| 66 | FLIP 09-326C | Kabuli | Syria |
| 67 | FLIP 09-329C | Kabuli | Syria |
| 68 | FLIP 09-330C | Kabuli | Syria |
| 69 | FLIP 09-332C | Kabuli | Syria |
| 70 | FLIP 09-333C | Kabuli | Syria |
| 71 | FLIP 09-334C | Kabuli | Syria |
| 72 | FLIP 09-335C | Kabuli | Syria |
| 73 | FLIP 09-336C | Kabuli | Syria |
| 74 | FLIP 09-337C | Kabuli | Syria |
| 75 | FLIP 09-338C | Kabuli | Syria |
| 76 | FLIP 09-341C | Kabuli | Syria |
| 77 | FLIP 09-342C | Kabuli | Syria |
| 78 | FLIP 09-344C | Kabuli | Syria |
| 79 | FLIP 09-345C | Kabuli | Syria |
| 80 | FLIP 09-347C | Kabuli | Syria |
| 81 | FLIP 09-350C | Kabuli | Syria |
| 82 | FLIP 09-351C | Kabuli | Syria |
| 83 | FLIP 09-352C | Kabuli | Syria |
| 84 | FLIP 09-353C | Kabuli | Syria |
| 85 | FLIP 09-354C | Kabuli | Syria |
| 86 | FLIP 09-355C | Kabuli | Syria |
| 87 | FLIP 09-356C | Kabuli | Syria |
| 88 | FLIP 09-358C | Kabuli | Syria |
| 89 | FLIP 09-359C | Kabuli | Syria |
| 90 | FLIP 09-361C | Kabuli | Syria |
| 91 | FLIP 09-362C | Kabuli | Syria |
| 92 | FLIP 09-363C | Kabuli | Syria |
| 93 | FLIP 09-366C | Kabuli | Syria |
| 94 | FLIP 09-367C | Kabuli | Syria |
| 95 | FLIP 09-370C | Kabuli | Syria |
| 96 | FLIP 09-371C | Kabuli | Syria |
| 97 | FLIP 09-372C | Kabuli | Syria |
| 98 | FLIP 09-374C | Kabuli | Syria |
| 99 | FLIP 09-377C | Kabuli | Syria |
| 100 | FLIP 09-378C | Kabuli | Syria |
| 101 | FLIP 09-386C | Kabuli | Syria |
| 102 | FLIP 09-387C | Kabuli | Syria |
| 103 | FLIP 09-388C | Kabuli | Syria |
| 104 | FLIP 09-389C | Kabuli | Syria |
| 105 | FLIP 09-391C | Kabuli | Syria |
| 106 | FLIP 09-392C | Kabuli | Syria |
| 107 | FLIP 09-394C | Kabuli | Syria |
| 108 | FLIP 09-396C | Kabuli | Syria |
| 109 | FLIP 09-397C | Kabuli | Syria |
| 110 | FLIP 09-399C | Kabuli | Syria |
| 111 | FLIP 09-400C | Kabuli | Syria |
| 112 | FLIP 09-401C | Kabuli | Syria |
| 113 | FLIP 09-403C | Kabuli | Syria |
| 114 | FLIP 09-404C | Kabuli | Syria |
| 115 | FLIP 09-405C | Kabuli | Syria |
| 116 | FLIP 09-408C | Kabuli | Syria |
| 117 | FLIP 09-409C | Kabuli | Syria |
| 118 | FLIP 09-411C | Kabuli | Syria |
| 119 | FLIP 09-412C | Kabuli | Syria |
| 120 | FLIP 09-416C | Kabuli | Syria |
| 121 | FLIP 09-423C | Kabuli | Syria |
| 122 | FLIP 09-425C | Kabuli | Syria |
| 123 | FLIP 09-430C | Kabuli | Syria |
| 124 | FLIP 09-437C | Kabuli | Syria |
| 125 | FLIP 09-438C | Kabuli | Syria |
| 126 | FLIP 2001-56C | Kabuli | Syria |
| 127 | FLIP 2003-127C | Kabuli | Syria |
| 128 | FLIP 94-62C | Kabuli | Syria |
| 129 | FLIP 97-210C | Kabuli | Syria |
| 130 | FLIP 97-230C | Kabuli | Syria |
| 131 | Genesis 090 | Kabuli | Australia |
| 132 | Genesis Kalkee | Kabuli | Australia |
| 133 | ICC 06098 | Desi | India |
| 134 | ICC 16796 | Kabuli | India |
| 135 | ICCV 91216 | Desi | India |
| 136 | ICCV 95224 | Desi | India |
| 137 | ICCV 96329 | Kabuli | India |
| 138 | IG131610C | Kabuli | India |
| 139 | Kimberly Large | Kabuli | Australia |
| 140 | Kyabra | Desi | Australia |
| 141 | NEC 02185C | Desi | India |
| 142 | PANT G-114C | Desi | India |
| 143 | PBA Monarch | Kabuli | Australia |
| 144 | PBA Pistol | Desi | Australia |
| 145 | PBA Seamer | Desi | Australia |
| 146 | PBA Slasher | Desi | Australia |
| 147 | S98477 | Kabuli | India |
| 148 | ICC 01671 | Kabuli | India |

**Appendix 3**. Pre-planting field preparations and continued field management

|  | **Narrabri** | **Kununurra** |
| --- | --- | --- |
| **Starter** | Granulock Z Extra at 80kg/Ha | 200 kg/ha DAP, 100 kg/ha SOP, 44 kg/ha Sulphur 90, Zinc monosulphate 10 kg/ha |
| **Inoculum** | TagTeam® peat | None |
| **sowing depth** | 9cm | 9cm |
| **Seed treatment** | P-Pickle T | P-Pickle T |
