# Supplementary figures and images for: A growing degree day model determines the effect of temperature stress on diverse chickpea genotypes
Source: Front Plant Sci. 2025 Feb 12;15:1496629. doi: 10.3389/fpls.2024.1496629 (PMC11861096; doi:10.3389/fpls.2024.1496629)

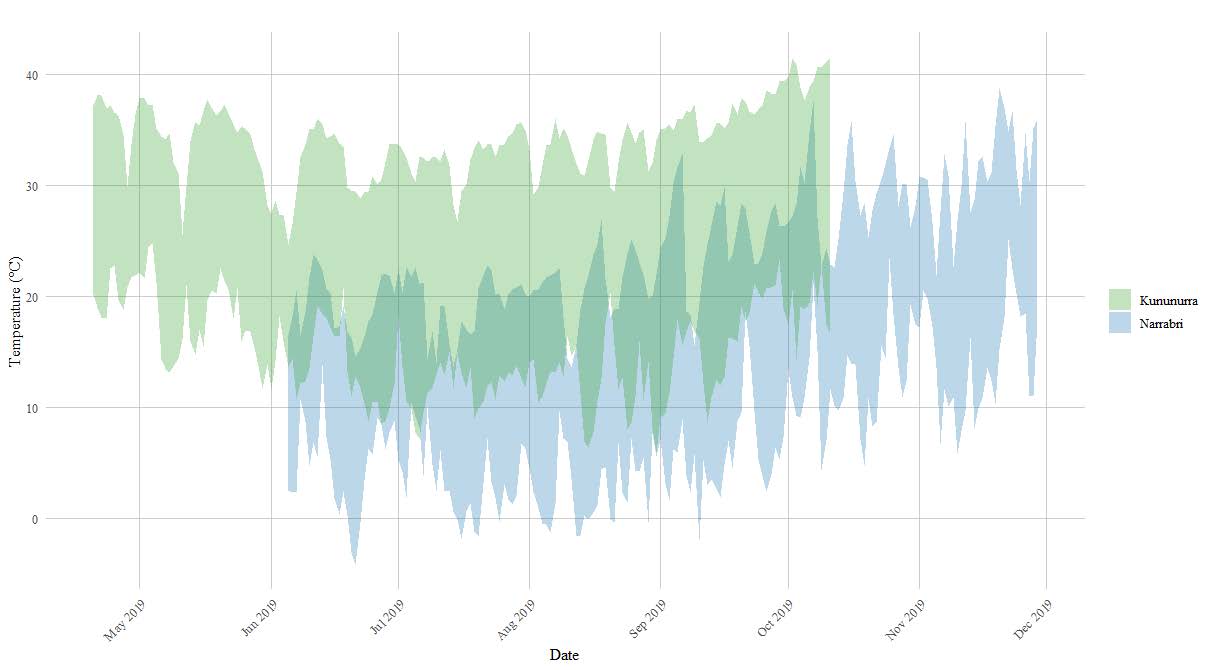

Supplement: Supplementary file 2 [file Image1.jpg]
